# A FREEZE IN TIME: PERCEPTION AND EXPERIENCE OF AMERICAN AND HONG KONG CHINESE OLDER ADULTS

**DOI:** 10.1093/geroni/igz038.2753

**Published:** 2019-11-08

**Authors:** Tinky Oi Ting Ho, Helene Hoi-Lam Fung, Vivian Hiu Ling Tsang, Angel Yee-lam Li, David J Ekerdt, Hansol Kim

**Affiliations:** 1 The Chinese University of Hong Kong, Hong Kong, Hong Kong; 2 The University of Hong Kong, Hong Kong, Not Applicable, Hong Kong; 3 The University of Kansas, Lawrence, Kansas, United States

## Abstract

According to self-continuity model, older adults are less likely to distinguish between the present and future, relative to younger adults. This mixed method design study aims at examining whether older adults perceive future as an infinite extension of present (i.e. “time freeze”) and investigating whether it is associated with life satisfaction, perceived control and perceived changes in future. 30 older adults from the US (aged 60-85, M = 78.4) and Hong Kong (aged 60-85, M =71.4) completed a structured interview and a survey. Findings revealed that 43% of Americans and 83% of Hong Kongers were experiencing ‘time freeze’. Individuals with a lower level of time freeze held more vivid and positive images of the future, and were achieving life goals actively, whereas individuals with a higher level of time freeze had comparatively more vague and neutral future views, and focused more on maintaining the current lifestyle.

